# Annual acknowledgement of manuscript reviewers

**DOI:** 10.1186/1472-6831-14-11

**Published:** 2014-02-10

**Authors:** Magdalena Morawska

**Affiliations:** 1 BioMed Central, Floor 6, 236 Gray’s Inn Road, London WC1X 8HB, UK

## Abstract

**Contributing reviewers:**

The editors of *BMC Oral Health* would like to thank all of our reviewers who have contributed to the journal in Volume 13 (2013).

## 

Abbas Azari

Iran

Abiodun Arigbede

Nigeria

Abiola Adeniyi

Nigeria

Akhilesh Sharma

India

Akihiro Yoshida

Japan

Alexander Rahman

Germany

Alina Puriene

Lithuania

Allan Pau

Malaysia

Alpdogan Kantarci

USA

Alvin Wee

USA

Amit Arora

Australia

Ana Mafla

Colombia

Anahita Jablonski-Momeni

Germany

Andreas Hellak

Germany

Anna Carolina Borges Pereira Costa

Germany

Anna-Lena Ostberg

Sweden

Anne Koerber

USA

Anne Nordrehaug Astrom

Norway

Anneli Lönnblad

Sweden

Arjen Van Wijk

Netherlands

Arnaldo Caldas Junior

Brazil

Atsuro Yokoyama

Japan

Atsushi Kameyama

Japan

Azam Bakhshandeh

Denmark

Bart Keijser

Netherlands

Bassam Hassan

Netherlands

Bella Monse

Philippines

Bjorn Axtelius

Sweden

Cacio Moura-Netto

Brazil

Carlos Quinonez

Canada

Carlos Estrela

Brazil

Carlos Moreira

Brazil

Carolina Borges

Brazil

Caterina Signoretto

Italy

Catherine Hong

Singapore

Catherine Binkley

USA

Celso Caldeira

Brazil

Chaiana Piovesan

Brazil

Charles Rwenyonyi

Uganda

Chengfei Zhang

Hong Kong

Chengzhang Li

China

Chiara Pirani

Italy

Christophe Bedos

Canada

Chun-Hung Chu

Hong Kong

Cibele Masotti

Brazil

Clarissa Bonifacio

Netherlands

Clovis Bramante

Brazil

Constantine Oulis

Greece

Cornelis De Putter

Netherlands

Cristiane Bendo

Brazil

Cristina Kurachi

Brazil

Daniele Manfredini

Italy

David Coleman

Ireland

David Bearn

UK

Demetrios Halazonetis

Greece

Dien Gambon

Netherlands

Dimitrios Ioannides

Greece

Dong Hun Han

South Korea

Dorthe Holst

Norway

Eduardo Moffa

Brazil

Edwin Ongkosuwito

Netherlands

Efigênia Ferreira

Brazil

Ei Wakamatsu

Japan

Eiichi Morii

Japan

Eino Honkala

Kuwait

Elizabeth Oziegbe

Nigeria

Elizangela Zuza

Brazil

Elsa Delgado

UK

Emelie Stenberg

Sweden

Emmanuel Silva

Brazil

Fabricio Zanatta

Brazil

Fabricio Collares

Brazil

Fawad Javed

Saudi Arabia

Frederico Neves

Brazil

Gerry Humphris

UK

Ghassan Matar

USA

Giovanna Batoni

Italy

Glenn Walker

Australia

Guglielmo Campus

Italy

Hans Ulrich Luder

Switzerland

Haroula Koletsi-Kounari

Greece

Helen Whelton

Ireland

Heliton Spindola Antunes

Brazil

Hidenobu Senpuku

Japan

Hiroyasu Endo

Japan

Hisatomo Kondo

Japan

Howard Bailit

USA

Ian Needleman

UK

Ichizo Morita

UK

Ilken Kocadereli

Turkey

Ingegerd Johansson

Sweden

Itzhak Brook

USA

Jacques-Henri Torres

France

James Deschner

Germany

James Crall

USA

Jan Clarkson

UK

Jan Mulder

Netherlands

Jason Armfield

Australia

Javier Caviedes

Colombia

Jefferson Traebert

Brazil

Jessica Lee

USA

Jimoh Olubanwo Agbaje

Nigeria

Jo Frencken

Netherlands

John Preisser

USA

Jonathan Broadbent

New Zealand

Jordi Caballé-Serrano

Switzerland

Jorma Virtanen

Finland

Jose Imparato

Brazil

Jose Leopoldo Ferreira Antunes

Brazil

Joyce Rose Masalu

Tanzania

Judith Raber-Durlacher

Netherlands

Juliana Stuginski-Barbosa

Brazil

Kabunda Syebele

South Africa

Kate Morgaine

New Zealand

Katsuyuki Kozai

Japan

Kazuyuki Ishihara

Japan

Keith Hunter

UK

Ken Kikuchi

Japan

Kerstin Öhrn

Sweden

Kimon Divaris

USA

Kostas Kapellas

Australia

Lars Bjorkman

Norway

Leonard Crocombe

Australia

Lilian Pozzer Ardenghi

Canada

Lin Zeng

USA

Linda Slack-Smith

Australia

Lin-P’Ing Choo-Smith

Canada

Lisa Heaton

USA

Lisa Christensen

Denmark

Luciana Shaddox

USA

Lucio Gonçalves

Brazil

Lyndie Foster Page

New Zealand

Magnus Hakeberg

Sweden

Majeed Rana

Germany

Malinee Laopaiboon

Thailand

Malka Ashkenazi

Israel

Manabu Morita

Japan

Marc Tennant

Australia

Marc Heft

USA

Marcelo Bortoluzzi

Brazil

Marcia Mayer

Brazil

Marco Peres

Australia

Marcos Pascoal Pattussi

Brazil

Maria Beatriz Gaviao

Brazil

Maria Do Carmo Freire

Brazil

Maria Letícia Ramos-Jorge

Brazil

Maria Regina Simionato

Brazil

Mariana Braga

Brazil

Mariana Marquezan

Brazil

Mariana Vizzotto

Brazil

Mario Tanomaru-Filho

Brazil

Mario Taba

Brazil

Matthew Hopcraft

Australia

Mevlut Celikoglu

Turkey

Michael Martin

USA

Michele D’Attilio

Italy

Michele Diniz

Brazil

Miha Bobic

Slovenia

Miira M Vehkalahti

Finland

Mike Morgan

Australia

Mireya Felhazy

USA

Mitsuo Kishi

Japan

Mohammad Khami

Iran

Mojtaba Dorri

Iran

Morenike Folayan

Nigeria

Muhammad Soyfoo

Belgium

Nagihan Bostanci

Switzerland

Nao Suzuki

Japan

Nazeer Khan

Pakistan

Newton Sesma

Brazil

Nikolaos Pandis

Greece

Nikolaos Gkantidis

Switzerland

Norman Tinanoff

USA

Pelin Guneri

Turkey

Paul Edwards

USA

Paul Allison

Canada

Paul Batchelor

UK

Paul Brunton

UK

Paul Aveyard

UK

Peggy Leong

USA

Peter Robinson

UK

Peter Murray

USA

Peter Bottenberg

Belgium

Peter Milgrom

USA

Piotr Fudalej

Switzerland

Pirkko Pussinen

Finland

Purnima Kumar

USA

Rachel Rocha

Brazil

Raluca Cosgarea

Germany

Raman Bedi

UK

Ramesh Nagarajappa

India

Rathna Devi Vaithilingam

Malaysia

Ratilal Lalloo

Australia

Rebecca Wassall

UK

Reinhard Gruber

Switzerland

Renata Mattos-Graner

Brazil

Richard Watt

UK

Riina Richardson

UK

Rita Shiang

USA

Robert Kerstein

USA

Robert Wright

USA

Rodrigo Souza

Brazil

Rodrigo Cunha

Canada

Rogerio Jacinto

Brazil

Roland Frankenberger

Germany

Roopali Sankeshwari

India

Roos Leroy

Belgium

Saman Warnakulasuriya

UK

Sandra Decker

USA

Sangeeta Palaskar

India

Sang-Joon Ahn

USA

Santosh Kumar Tadakamadla

India

Saul Paiva

Brazil

Sebastian Hahnel

Germany

Seung-Hak Baek

South Korea

Sharat Chandra Pani

Saudi Arabia

Sharon Liberali

Australia

Sigrid Van Den Branden

Belgium

Silvio Abati

Italy

Sladjana Malic

UK

Sonica Singhal

Canada

Stefan Ruhl

USA

Stefania Martignon

Colombia

Stephen Rosenstiel

USA

Sudaduang Krisdapong

Thailand

Suguru Kimoto

Japan

Suk-Ja Yoon

South Korea

Susan Bridges

China

Susan Muller

USA

Svetlana Tikhonova

Canada

Takahiko Oho

Japan

Takuichi Sato

Japan

Tamara Tedesco

Brazil

Tamer Tasdemir

Turkey

Tatiana Pereira-Cenci

Brazil

Tegwyn Brickhouse

USA

Tewarit Somkotra

Thailand

Thiago Ardenghi

Brazil

Thomas Thurnheer

Switzerland

Thorakkal Shamim

India

Tim Newton

UK

Tonnie Mulli

Kenya

Toshihiro Ansai

Japan

Trilby Coolidge

USA

Ugur Inan

Turkey

Ulvi Gursoy

Finland

Vanessa Muirhead

UK

Wael Sabbah

UK

Wei Seong Toh

Singapore

Wil Van Der Sanden

Netherlands

William Murray Thomson

New Zealand

Xiao-Wen Jiang

China

Yasuhiko Kawai

Japan

Young J Kim

South Korea

Yu-Kang Tu

UK

Zoe Marshman

UK

## Pre-publication history

The pre-publication history for this paper can be accessed here:

http://www.biomedcentral.com/1472-6831/14/11/prepub

